# Epidemiology and Clinical Features of Ciguatera Fish Poisoning in Hong Kong

**DOI:** 10.3390/toxins6102989

**Published:** 2014-10-20

**Authors:** Thomas Y. K. Chan

**Affiliations:** 1 Division of Clinical Pharmacology and Drug and Poisons Information Bureau, Department of Medicine and Therapeutics, Faculty of Medicine, the Chinese University of Hong Kong, Prince of Wales Hospital, Shatin, New Territories, Hong Kong, China; 2 Centre for Food and Drug Safety, Faculty of Medicine, the Chinese University of Hong Kong, Hong Kong, China; E-Mail: tykchan@cuhk.edu.hk; Tel.: +852-2632-3907; Fax: +852-2646-8756

**Keywords:** ciguatera, ciguatoxins, groupers, Hong Kong

## Abstract

In the present review, the main objective was to describe the epidemiology and clinical features of ciguatera fish poisoning in Hong Kong. From 1989 to 2008, the annual incidence of ciguatera varied between 3.3 and 64.9 (median 10.2) per million people. The groupers have replaced the snappers as the most important cause of ciguatera. Pacific-ciguatoxins (CTX) are most commonly present in reef fish samples implicated in ciguatera outbreaks. In affected subjects, the gastrointestinal symptoms often subside within days, whereas the neurological symptoms can persist for weeks or even months. Bradycardia and hypotension, which can be life-threatening, are common. Treatment of ciguatera is primarily supportive and symptomatic. Intravenous mannitol (1 g/kg) has also been suggested. To prevent ciguatera outbreaks, the public should be educated to avoid eating large coral reef fishes, especially the CTX-rich parts. A Code of Practice on Import and Sale of Live Marine Fish for Human Consumption for Prevention and Control of Ciguatera Fish Poisoning was introduced from 2004 to 2013. The Food Safety Ordinance with a tracing mechanism came into full effect in February 2012. The Government would be able to trace the sources of the fishes more effectively and take prompt action when dealing with ciguatera incidents.

## 1. Introduction

Ciguatera fish poisoning is caused by consumption of tropical and subtropical coral reef fishes that have bioaccumulated ciguatoxins (CTX). Typically, these are large predator fishes such as moray eels, snappers, groupers, Spanish mackerels and barracuda [1]. CTX originate in the toxic dinoflagellates of the *Gambierdiscus* species found on coral reefs and undergo bioaccumulation and biotransformation as they pass through the food chain [2]. CTX are mainly found in Pacific (P-CTX), Caribbean (C-CTX) and Indian Ocean (I-CTX) regions; they differ in potency (P-CTX > I-CTX > C-CTX) as activators of voltage-sensitive sodium channels in excitable membranes of nerves and muscles [1,3]. The structures and characteristics of P-CTX, I-CTX and C-CTX are described in detail elsewhere [4]. CTX directly stimulate intestinal fluid secretion [1]. Autonomic dysfunction leading to hypotension and bradycardia also occurs [5]. As CTX are heat-stable and lipid-soluble, cooking offers no protection and ingestion of large coral reef fishes (especially the CTX-rich parts—head, viscera, roe and skin) can cause severe illness and prolonged symptoms [6,7]. Ciguatera is characterized by a combination of gastrointestinal, neurological and cardiovascular signs and symptoms [3,6,7]. Gastrointestinal symptoms (e.g., nausea, vomiting, abdominal pain and diarrhea) are self-limiting. Cardiovascular features (sinus bradycardia and hypotension) can be life-threatening but respond to intravenous fluids, atropine and dopamine [8]. Neurological symptoms (e.g., paresthesia/numbness of lips, tongue and the four limbs, reversal of hot-cold sensation, myalgia, muscle weakness, arthralgia, pruritus and fatigue) are also common and can last for weeks or even months (e.g., paresthesia/numbness of four limbs and fatigue).

The global incidence and geographical distribution of ciguatera are on the increase [9]. Ciguatera is endemic in tropical and subtropical regions of the Pacific, Caribbean and Indian Ocean [10]. However, with increases in international tourism and reef fish trade, cases are frequently reported in non-endemic regions as well [9]. Under-diagnosis and particularly under-reporting of ciguatera illness are common [9]. In view of its medical, social and economic importance [1,3,7,9], more efficient data collection, at the global, regional and national levels, on the occurrence and clinical manifestations of ciguatera will be necessary to facilitate the planning of preventive strategies and management of patients [6,7].

In the present review, the main objective was to describe the epidemiology and clinical features of ciguatera fish poisoning in Hong Kong from 1988 to 2008, based on all available data.

## 2. Published Reports and Statistics on Ciguatera

To identify published articles in indexed journals and non-indexed journals, a search of the Medline (1985 to 22 August 2014) and Google Scholar was performed, using ciguatera poisoning, ciguatoxins and Hong Kong as the search terms. Additional papers were identified from the article database of the Drug and Poisons Information Bureau (http://dpib.med.cuhk.edu.hk/).

Other relevant publications, press releases and local information on ciguatera were identified from the websites of Centre for Food Safety (http://www.cfs.gov.hk/eindex.html) and Centre for Health Protection (http://www.chp.gov.hk/en/), Department of Health, The Government of the Hong Kong Special Administration Region. In Hong Kong, food poisoning including ciguatera is a statutory notifiable disease and notification of suspected or confirmed cases is required by law.

## 3. First Reports of Ciguatera in Hong Kong

As indicated by the early review by Department of Health [11], ciguatera was first reported in Hong Kong in 1988. The territory-wide Drug and Poisons Information Bureau [12] first received reports of ciguatera in 1991–1992. In the first outbreak [13], nine people became ill after eating a mangrove snapper (*Lutjanus argentimaculatus*) and two required hospital admission. In the second outbreak [8], four subjects fell sick after eating a mangrove snapper and three required hospital admission. Sporadic cases as well as large outbreaks of ciguatera were known to occur since.

## 4. Incidence of Ciguatera and the Fish Species Responsible

Figures on the number of outbreaks and total number of subjects affected per year were provided by Centre for Health Protection, Department of Health [14,15,16]. The mid-year population of Hong Kong (Census and Statistics Department) was used to calculate the annual incidence per million people.

**Figure 1:**
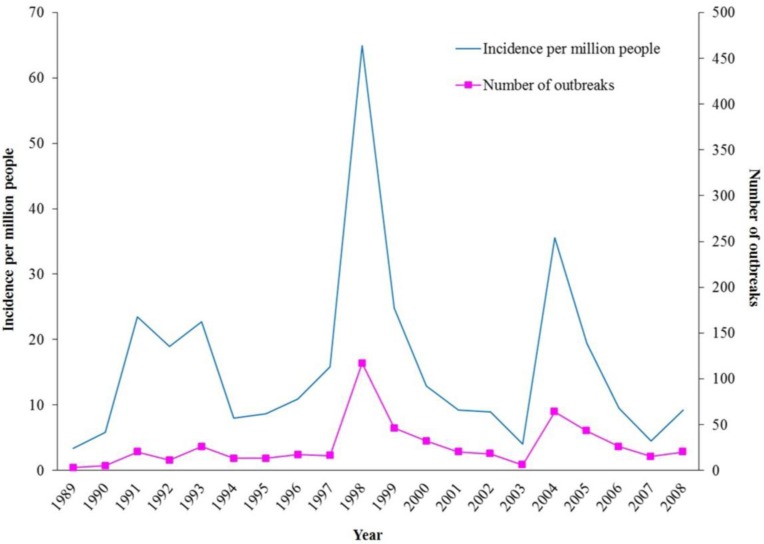
Annual figures on ciguatera, 1989–2008. (Data from Department of Health).

During the 20-year period in 1989–2008, there were three to 117 outbreaks (median 19), affecting 19 to 425 subjects (median 68) each year. The annual incidence of ciguatera varied between 3.3 and 64.9 (median 10.2) per million people, with two peaks in 1998 and 2004 (64.9 and 35.5 per million people) (Figure 1).

**Figure 2:**
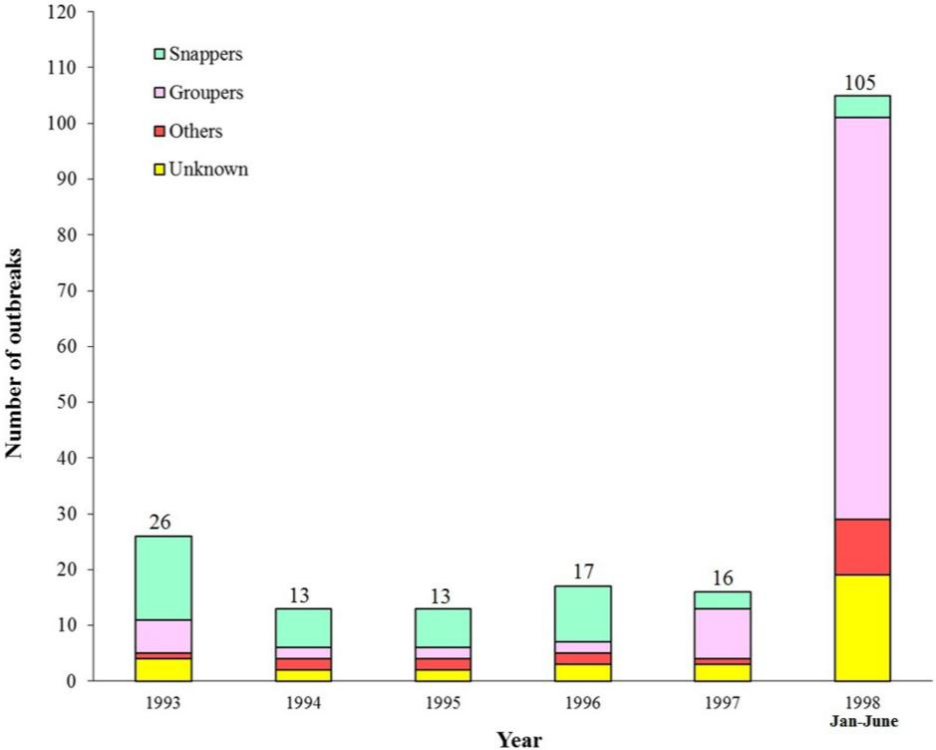
Fish species causing ciguatera outbreaks, 1993–1998. (Data from Department of Health).

From 1988 to 1992, the snappers and the groupers accounted for 28 (59.6%) and 15 (31.9%) of 47 outbreaks of ciguatera [11]. Data from the Department of Health [17] and the subsequent reports [7,15,16] indicated the dominance of the groupers as from 1997 (Figure 2).

## 5. Ciguatoxins Responsible

In Hong Kong, both mouse bioassay (MBA) and liquid chromatography-tandem mass spectrometry (LC-MS/MS) are used by the Public Health Laboratory Services Branch, Centre for Health Protection, Department of Health to identify and quantify CTX present in reef fish samples implicated in ciguatera outbreaks [15,18,19,20]. In their retrospective analysis of 27 MBA-positive fish samples (groupers = 16, snappers = 9, others = 2) collected during 2004 to 2013 [20], P-CTX-1, P-CTX-2 and P-CTX-3 were most commonly present, confirming the Pacific Ocean region as the most important origin of ciguatera fish poisoning. This may explain the predominance of neurological features in the affected individuals (see section 6). For practical application, a two-tiered approach has been suggested—chemical analysis by LC-MS/MS for screening of CTX in potentially toxic reef fish in the market, coupled with bioassay by MBA for final toxicity confirmation [20]. Their aim is to minimize the use of laboratory mice, while providing a reasonably effective means for routine screening of CTX in reef fish [20].

## 6. Clinical Manifestations and Notable Cases

The three largest published case series of ciguatera [11,15,21] are reviewed to characterize the frequency and types of clinical manifestations (Table 1). Two large case series [11,16] were notifications received by the Department of Health from the whole of Hong Kong during 1988–1992 and January 2004–May 2005. The third case series [21] were adults receiving treatment in three general hospitals during 2003–2006 [6,7,22,23,24].

**Table 1:** Incubation period and clinical manifestations of ciguatera (incidence, %).

	Choi *et al*. [11] ^a^	Au [16] ^a^	Chan *et al*. [21] ^b^
	1988–1992 (*n* = 397)	1993–2005 (*n* = 300)	2003–2006 (*n* = 18) ^c^
**Incubation period (range, median)**	1–30 h (7.5 h)	0.25–43 h (5.5 h)	1–7 h
**Gastrointestinal**			
Nausea	29	40	61
Vomiting	22	31	78
Abdominal pain	47	59	56
Diarrhea	58	74	94
**Neurological**			
Paresthesia/numbness (4 limbs)	78	79	67
Paresthesia/numbness (perioral/tongue/face)	30	54	50
Muscle weakness	44	54	50
Myalgia	24	- ^e^	22
Headache	19	19	0
Hot/cold reversal	16	19	6
Dizziness ^d^	- ^e^	37	61
**Cardiovascular**			
Bradycardia	0.3	- ^e^	94
Hypotension	0.3	- ^e^	94
**General**			
Fatigue	59	- ^e^	6
Pruritus	11	- ^e^	6
Skin rash	2	- ^e^	0

^a^ Phone interview; ^b^ hospital notes reviews and ^a^ questionnaires used to collect data might affect the rates; ^c^ Ataxia (*n* = 3); ^d^ Dizziness could be due to bradycardia and hypotension; ^e^ Symptoms/signs not listed.

Affected individuals generally developed a combination of gastrointestinal, neurological and other symptoms and signs (Table 1). The gastrointestinal symptoms often subsided within days, whereas the neurological symptoms (e.g., paresthesia/numbness of the four limbs) could persist for weeks or even months [11,16]. In contrast to hospital-based reviews [21], phone interviews [11,16] might not identify bradycardia and hypotension. These cardiovascular complications were much more common (94%) in 2003–2006. In 17 subjects [21], ECG revealed sinus bradycardia (*n* = 15), junctional bradycardia (*n* = 1) or Wenckebach second-degree AV block (*n* = 1).

In addition, three cases should be briefly mentioned here in view of their life-threatening cardiovascular features and chronicity of neurological symptoms. A 50-year-old man developed acute gastrointestinal and neurological symptoms after the consumption of an unidentified coral reef fish head [24]. He also developed dizziness, severe bradycardia (46 beats per minute) and prolonged hypotension. He needed ICU care, administration of intravenous atropine and over three days of intravenous fluid replacement with dopamine infusion. A 40-year-old man experienced ciguatera symptoms after eating some coral reef fish [25]. The gastrointestinal symptoms and headache subsided in 1 week; the neurological symptoms and arthralgia persisted for 1 month. Two years later, after drinking 300 mL of beer, he experienced a relapse of neurological symptoms and arthralgia, which subsided within 1 week. A 43-year-old lady developed ciguatera with marked motor symptoms after eating a grouper (*Epinepheius* spp.) [26]. Her muscle weakness in the four limbs lasted over 45 days. Chronicity of neurological features might indicate a lengthy persistence of CTX in the body [25,26].

## 7. Diagnosis and Treatment

The diagnosis of ciguatera is mainly based on epidemiological evidence and clinical grounds, with a history of reef fish consumption followed by the typical clinical features (Table 1). Fish remnants may not be available for identification of CTX.

Treatment of ciguatera is primarily supportive and symptomatic. Dehydration and hypovolemia as a result of vomiting and diarrhea should be corrected. Severe bradycardia and prolonged hypotension can occur, necessitating prompt treatment with intravenous atropine, intravenous fluid replacement and dopamine infusion [21,24]. Intravenous mannitol (1 g/kg body weight over 1 h) within 24 h of symptom onset has been suggested because of the marked improvements in the neurological symptoms seen in some patients [23,27]. However, a double-blind, randomized controlled study of 50 patients in Rarotonga, Cook Islands revealed that normal saline was as effective as mannitol in relieving ciguatera symptoms at 24 h [28].

## 8. Prevention

To prevent ciguatera outbreaks, the public should be educated to avoid eating large coral reef fishes, especially the CTX-rich parts—head, viscera, roe and skin [6,7]. Avoidance of fishes in excess of 2 kg is generally recommended [7,11], but occasionally fishes weighing 0.6 kg or less were also implicated [29]. Information on the potential toxic fish species, local ciguatera situation and preventive measures are available in the website of Centre for Food Safety (http://www.cfs.gov.hk/eindex.html).

In Hong Kong, food poisoning including ciguatera is a statutory notifiable disease. Suspected cases are investigated by Centre for Health Protection and Centre for Food Safety [11,16,29]. Investigations aim to collect essential information for assessment of impact and planning of preventive measures (e.g., epidemiological and clinical features, types and origins of reef fishes, and quantities of CTX involved). Early control of a possible ciguatera outbreak will also be possible, mainly through publicity measures (press releases) to promote awareness, public education and avoidance of high risk fish [29,30].

Centre for Food Safety repeatedly advises the trade to avoid sourcing fishes from high-risk regions [29]. In addition, a Code of Practice on Import and Sale of Live Marine Fish for Human Consumption for Prevention and Control of Ciguatera Fish Poisoning was introduced from 2004 to 2013 [30]. After the Food Safety Ordinance (Chapter 612) with a tracing mechanism came into full effect in February 2012 [31], all food traders must keep relevant transaction records. The Government would now be able to trace the sources of the fishes more effectively and take prompt action when dealing with ciguatera incidents.

## 9. Conclusions

In Hong Kong, sporadic cases, as well as large outbreaks of ciguatera have been known to have occurred since 1988. From 1989 to 2008, the annual incidence of ciguatera varied between 3.3 and 64.9 (median 10.2) per million people, with two peaks in 1998 and 2004 (64.9 and 35.5 per million people). The groupers have replaced the snappers as the most prevalent cause of ciguatera. P-CTX are most commonly present in reef fish samples implicated in ciguatera outbreaks. In affected subjects, the gastrointestinal symptoms often subside within days, whereas the neurological symptoms (e.g., paresthesia/numbness of the four limbs) can persist for weeks or even months. Bradycardia and hypotension, which can be life-threatening, are much more common since 2003. As fish remnants may not be available for identification of CTX, the diagnosis of ciguatera is mainly based on epidemiological evidence and clinical grounds. Dehydration and hypovolemia, if present, should be corrected. If severe bradycardia and prolonged hypotension occur, prompt treatment with intravenous atropine, intravenous fluid replacement and dopamine infusion will be necessary. Intravenous mannitol (1 g/kg) within 24 h of symptom onset has also been suggested because of the marked improvements in the neurological symptoms seen in some patients.

To prevent ciguatera outbreaks, the public should be educated to avoid eating large coral reef fishes, especially the CTX-rich parts. Information on the potential toxic fish species, the local ciguatera situation and preventive measures are available in the website of the Centre for Food Safety. A Code of Practice on Import and Sale of Live Marine Fish for Human Consumption for Prevention and Control of Ciguatera Fish Poisoning was introduced from 2004 to 2013. After the Food Safety Ordinance (Chapter 612) with a tracing mechanism came into full effect in February 2012, the Government would now be able to trace the sources of the fishes more effectively and take prompt action when dealing with ciguatera incidents.
